# Novel Species of *Brucella* Causing Human Brucellosis, French Guiana

**DOI:** 10.3201/eid2902.220725

**Published:** 2023-02

**Authors:** Frédégonde About, Theo Pastre, Mathilde Boutrou, Alex Yahiaoui Martinez, Alessia Melzani, Sandrine Peugny, Céline Michaud, Sami Zouaoui, Thierry Carage, Vincent Sainte Rose, Magalie Demar, Jean-Philippe Lavigne, Félix Djossou, David O’Callaghan, Loïc Epelboin, Anne Keriel

**Affiliations:** Centre Hospitalier de Cayenne Andrée Rosemon, Cayenne, French Guiana (F. About, M. Boutrou, A. Melzani, C. Michaud, V. Sainte Rose, M. Demar, F. Djossou, L. Epelboin);; Centre Hospitalier de Kourou, Kourou, French Guiana (F. About, M. Boutrou, S. Zouaoui, T. Carage);; Carémeau University Hospital de Nimes, Nimes, France (T. Pastre, A.Y. Martinez, J.-P. Lavigne, D. O’Callaghan, A. Keriel);; Centre Hospitalier de l’Ouest Guyanais, Saint-Laurent du Maroni, French Guiana (S. Peugny);; Université de Guyane, Cayenne (M. Demar, L. Epelboin);; Université de Montpellier, Nimes (J.-P. Lavigne, D. O’Callaghan, A. Keriel);; Institut National de la Santé et de la Recherche Médicale (INSERM), Nimes (D. O’Callaghan, A. Keriel);; INSERM, Cayenne (L. Epelboin)

**Keywords:** *Brucella*, brucellosis, French Guiana, zoonoses, bacteria

## Abstract

Human brucellosis is a zoonoses caused by bacteria of the genus *Brucella*. Infection results in subacute or chronic debilitating disease with nonspecific clinical manifestations and is often associated with consuming unpasteurized dairy products. We report 2 cases of brucellosis in male patients who were hospitalized in distinct towns of French Guiana, an overseas territory of France located on the northeastern shore of South America. Both men were citizens of Brazil working as clandestine goldminers in the deep Amazonian rainforest. Characterization of the 2 bacterial isolates revealed that they represent a potential new species of *Brucella*. Medical practitioners working in contact with wildlife in this region of the world should be aware of the existence of these pathogens and the potential for human infection.

Human brucellosis is a subacute or chronic debilitating disease with nonspecific clinical manifestations. Presenting classically as an influenza-like syndrome, this zoonosis remains endemic in most developing countries, mainly in areas with extensive farming, and transmission is commonly traced to consumption of unpasteurized dairy products (*1*). Direct exposure to diseased animals can also transmit infection, and cases have been reported in persons who had prior contact with wild animals (*2*,*3*).

Brucellosis is caused by gram-negative, facultative intracellular bacteria of the *Brucella* genus that can infect many organs and soft tissues. For ≈40 years, only 6 *Brucella* species were known, including the 3 species most predominant in livestock and humans: *B. melitensis* (goats), *B. abortus* (cows), and *B. suis* (swine, reindeer, and hares). The past decade, however, has seen a rapid expansion of both known members of the *Brucella* genus and the variety of associated animal hosts, which now range from mammals to amphibians and fish (*4*). Recently described *Brucella* species include *B. ceti* (cetaceans), *B. pinnipedialis* (pinnipeds), *B. inopinata* (humans), *B. microti* (voles), *B. papionis* (baboons), and *B. vulpis* (foxes). Several *Brucella* isolates, identified in rodents, frogs, reptiles, fish, and bats, still await formal taxonomic description. 

Genomically, the *Brucella* genus is divided into classical species—*B. melitensis*, *B. abortus*, *B. suis*, *B. canis* (dog), *B. ovis* (sheep), *B. neotomae* (desert rats), *B. ceti*, *B. pinnipedialis*, *B. papionis,* and *B. microti*—and so-called atypical species. All *Brucella* are genetically closely related, showing genome similarities of >90 % at the nucleotide level. However, *Brucella* species can be clearly separated from each other by highly discriminating molecular techniques, such as multiplex PCR, multilocus sequencing typing, or multiple loci variable-number tandem repeat analysis.

French Guiana is an overseas region of France that is on the northeastern coast of South America and covered largely (95%) by the Amazon rainforest. A recent report described human infection in French Guiana with *B. suis* bv1, which was likely contracted from domestic pigs (A. Melzani et al., unpub. data). We report 2 cases of human brucellosis in 2 patients hospitalized in distinct towns of French Guiana. Genome analysis revealed that 2 bacterial isolates represent a potential new species of *Brucella*.

## Material and Methods

### Case Reports

**Figure 1:**
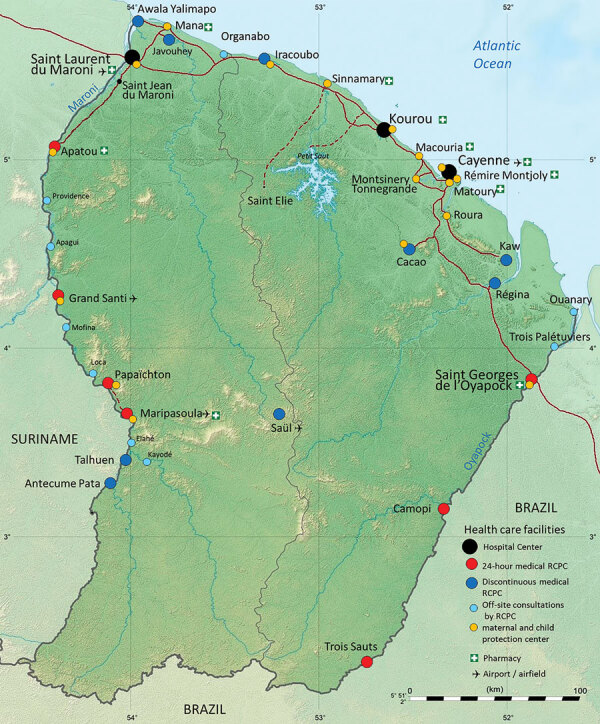
Locations of main towns and care facilities in French Guiana.

Patient 1 was a 39-year-old man, originally from Belem, Brazil, who made his living as a clandestine goldminer. He had lived in Suriname for 10 years and had arrived in French Guiana 8 days before the onset of his symptoms in Maripasoula, a town of ≈10,000 inhabitants on the Maroni River (Figure 1). He sought care at Maripasoula Health Center in early September 2020 for fever, asthenia, and lower back pain. He did not have a relevant medical history. Clinical examination found hepatomegaly. Biological analyses showed hemoglobin of 10.2 g/dL (reference range 13–18 g/dL), mean corpuscular volume 78 µm^3^ (reference range 80–100 µm^3^), leukocytes 3.9 g/L (reference range 4.5–11 g/L), platelets 184 g/L (reference range 150–400 g/L), C-reactive protein 47.9 mg/L (reference range 3–10 mg/L), aspartate aminotransferase 201 IU/L (reference range <40 IU/L), and alanine aminotransferase 187 IU/L (reference range <35 IU/L). HIV serology results were was negative. The patient was transferred to Cayenne Hospital on day 3 of empirical treatment, with a treatment course of intravenous ceftriaxone (1 g/d). Because of ongoing fever, antimicrobial therapy was switched to piperacillin/tazobactam. Computed tomography of the chest, abdomen, and pelvis showed isolated hepatomegaly (19 cm) and splenomegaly (13 cm). Gram-negative bacilli were identified in blood cultures on day 9, after 68 hours of incubation. Mass spectrometry (MALDI Biotyper [Bruker, https://www.bruker.com/en.html]; security-relevant database; score 2.4) and VITEK 2GN ID card (bioMerieux, https://www.biomerieux.fr) (score 98%) identified the presence of *B. melitensis*. No serodiagnosis could be performed. 

After establishing a diagnosis of brucellosis (September 15, 2020), we switched the patient’s antibiotic regimen to a combination of rifampin (900 mg 1×/d) plus oral doxycycline (100 mg 2×/d) and a dose of gentamicin (250 mg). Blood cultures were positive for *Brucella* species through September 21. Transthoracic echocardiography showed no evidence of endocarditis. The patient denied being exposed to farm animals or consuming unpasteurized milk and cheese but admitted to consuming game hunted in the forest around the goldmine camp, including wild pork meat. He was discharged and returned home, continuing antibacterial therapy for a total of 6 weeks. At the last consultation (after 1 month of antibiotics), his progress was satisfactory, but the patient was unavailable for further follow-up.

Patient 2 was a 45-year-old man from Macapá, Brazil, who was working as a blacksmith at an illegal goldmine near Apatou (Figure 1). He had been living on both the French Guiana and Surinam sides of the Maroni River since approximatly 2002. In October 2020, the patient sought treatment at the Hospital of Saint-Laurent du Maroni for fever, lower back pain, weight loss of ≈8 kg in 10 months, and functional impairment of his left leg. He had no relevant medical history. Clinical examination found pain during active mobilization of the left psoas. Testing ruled out neurologic disorders. Laboratory tests showed hemoglobin of 11.1 g/dL, mean corpuscular volume g 86 µm^3^, leukocytes of 5.4 g/L, platelets 274 g/L, and C-reactive protein 126.5 mg/L. HIV serology results were negative. Computed tomography of the lumbar spine revealed advanced spondylodiscitis of the L4–L5 interline line, anterior prevertebral collection and destruction of adjacent vertebral bodies, and posterior disc filling narrowing the spinal canal. Collection extended to the left psoas. The paravertebral collections measured 49 × 51 × 19 mm and a left psoas collection measured 95 × 39 × 42 mm. Magnetic resonance imaging of the lumbar spine revealed epidural inflammation and compression of the terminal cone by the intra-anal collection. Transthoracic echocardiography revealed no valvular disease and infective endocarditis. 

The patient was transferred to the orthopedic surgery unit of Kourou Hospital for psoas abscess sampling. He was treated empirically with antituberculosis drugs (rifampin 480 mg, isoniazid 200 mg, pyrazinamide 1,200 mg, and ethambutol 750 mg 1×/d) in combination with corticosteroids. *B. melitensis* was identified on culture, after 6 days of incubation, from a sample obtained from the psoas abscess. All blood culture results before administration of antibiotics were negative after 5 days of incubation. After a diagnosis of brucellosis, we changed the patient’s treatment to doxycycline (100 mg 2×/d) and rifampin (600 mg 1×/d), in combination with gentamicin (250 mg/d for 6 d). The patient did not declare being exposed to farm animals, but he did eat meat hunted in the forest. His initial clinical outcome was favorable, with a decrease of fever. However, the patient left the hospital (on day 18 of treatment) and before completion of treatment and was not available for follow-up.

### Molecular Typing of Bacteria

We extracted bacterial genomic DNA by using the DNeasy UltraClean Microbial Kit (QIAGEN, https://www.qiagen.com) according to the manufacturer’s instructions and quantified genomic DNA with a Qubit fluorimeter (ThermoFisher Scientific, https://www.thermofisher.com). We performed Bruce-Ladder and Suis-Ladder multiplex PCRs as described (*5*,*6*).

### Genome Sequencing and Analysis

We sequenced genomic DNA by using an Illumina MiSeq (Illumina, https://www.illumina.com) platform. We used a Nextera DNA Flex Kit (Illumina) for the library preparation, using 500 ng of DNA as input. We assessed library molarity and quality on the Qubit and the tape station using a DNA high-sensitivity chip (Agilent Technologies, https://www.agilent.com). We used the MiSeq Reagent Kit v2 (Illumina) to load libraries. Paired-end reads of 150 bp were generated de novo and assembled by using Shovill 1.1.0, a GALAXY webtool (*7*), and the contigs were assembled via the CONTIGuator web server, using the *B. ceti TE10759–12* genome (BioProject PRJNA224116) as a reference. We annotated consensus sequences by using the PATRIC genome annotation service (RASTtk) (*8*).

We deposited the genome sequences in GenBank under BioProject no. PRJNA728965 (accession nos. CP074683 for chromosome 1 and CP074684 for chromosome 2 of BRSO-2021–230 [BioSample SAMN19108195]; CP075601 for chromosome 1 and CP075602 for chromosome 2 of BRSO-2020–213 [BioSample SAMN19107925]).

## Results

### Microbiological Characterization

We shipped bacterial isolates from both patients to the French *Brucella* National Reference Center in Nimes, France, for confirmation of identification; they were named BRSO-2020–213 (Cayenne) and BRSO-2021–230 (Kourou). Analysis by matrix-assisted laser desorption/ionization time-of-flight mass spectrometry (Vitek MS with IVD database v3.2.0; bioMérieux) confirmed that both isolates belong to the *Brucella* genus. Slide agglutination using a polyclonal serum anti-*Brucella* (ANSES, https://www.anses.fr) also yielding positive results. Both isolates are gram-negative bacteria, forming nonhaemolytic, round, convex, smooth, greyish colonies with a diameter of ≈0.5 mm after 48 hours of incubation on tryptic soy agar. Both isolates showed positive reactions for catalase (slide test), cytochrome-oxidase and urease (analytical profile index gallery tests) and a negative result for indole production (Table).

**Table:** Results of gallery tests for *Brucella* isolates obtained from 2 patients with brucellosis, French Guiana, 2020

Incubation time	Test	BRSO-2020-213	BRSO-2021-230
24 h	Reduction of nitrates to nitrites or nitrogen	+	+
	Indole production	–	–
	Fermentation of glucose	–	–
	Arginine dihydrolase	–	–
	Urease	+	+
	Hydrolysis of esculin	–	–
	Hydrolysis of gelatin	–	–
	β-galactosidase production	–	–
48 h	Glucose assimilation	+	+
	Arabinose assimilation	+	+
	Mannose assimilation	+	+
	Mannitol assimilation	-	-
	N-acetyl-glucosamine assimilation	+	+
	Maltose assimilation	–	–
	Potassium gluconate assimilation	–	–
	Capric acid assimilation	–	–
	Adipic acid assimilation	–	–
	Malate assimilation	–	–
	Trisodium citrate assimilation	–	–
	Phenyl acetic acid assimilation	–	–
	Cytochrome oxidase	+	+

*API20NE gallery tests (bioMérieux, https://www.biomerieux.com) were performed according to the manufacturer’s instructions. +, positive, –, negative.

### Analysis of Bacterial Genomes

**Figure 2:**
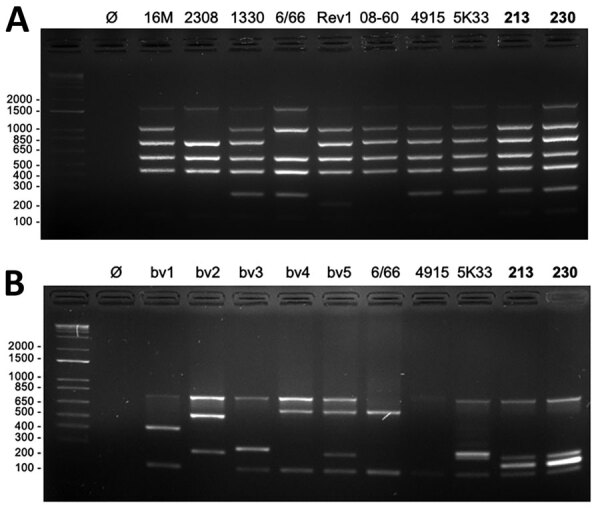
PCR typing of newly discovered *Brucella* isolates obtained from 2 patients in French Guiana, 2020**.** Genomic DNA from bacterial isolates BRSO-2020-213 and BRSO-2021-230 (numbers in bold) were used as a template for Bruce-Ladder (*5*) (A) or Suis-Ladder (*6*) (B) multiplex PCR. The profiles obtained after agarose gel electrophoresis were compared with the profile of type strains 16M (*Brucella melitensis*), 2308 (*B. abortus*), 1330 (*B. suis* bv1), Thomsen *(B. suis* bv2), 686 (*B. suis* bv3), 40 (*B. suis* bv4), 513 (*B. suis* bv5), RM6/66 (*B. canis*), Rev1 (*B. melitensis* vaccine strain), F8/08–60 (*B. papionis*), CCM 4915 (*B. microti*), or 5K33 (*B. neotomae*). Molecular-weight size markers in bp are shown at left. bv, biovar; Ø, negative control.

We further characterized the isolates at the genomic level using the Bruce-Ladder multiplex PCR (*5*). The profiles obtained suggested that both could belong to the species *B. suis*, *B. microti*, or *B. neotomae* (Figure 2, panel A). However, profiles derived by using the Suis-Ladder PCR, a multiplex PCR designed to discriminate between different biovars of *B. suis*, were not similar to any reported *Brucella* species (Figure 2, panel B) (*6*). We then performed whole-genome sequencing, which revealed that both genomes contain 2 chromosomes (Chr) with a size and guanine-cytosine content similar to other *Brucella* species (BRSO-2020-213: Chr1 = 2,104,069 pb, Chr2 = 1,192,383 pb, 57.25% guanine-cytosine; BRSO-2021-230: Chr1 = 2,097,320 pb, Chr2 = 1,191,781 bp, 57.26% guanine-cytosine). No plasmid was detected. Annotation of genomes revealed the presence of several genetic features that are specific to *Brucella*: the bcsp31 gene on Chr1, the *virB* operon (*9*) on Chr2, and several copies of the IS711 insertion sequence (*10*).

**Figure 3:**
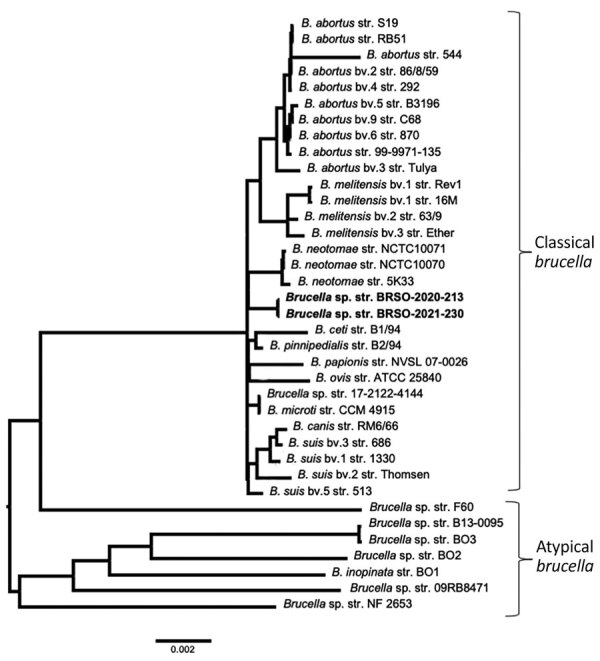
Phylogenetic placement of *Brucella* isolates BRSO-2020-213 and BRSO-2021-230 from 2 patients diagnosed with brucellosis in French Guiana, 2020. A genome-based tree was constructed by using a selection of reference *Brucella* genomes (Appendix Table) on the PATRIC platform (BRC NIAID Bioinformatics Resource Centers, https://www.bv-brc.org). The number of compared genes was fixed at 1,000, and all other parameters were set by default. Scale bar indicates number of substitutions per site.

**Figure 4:**
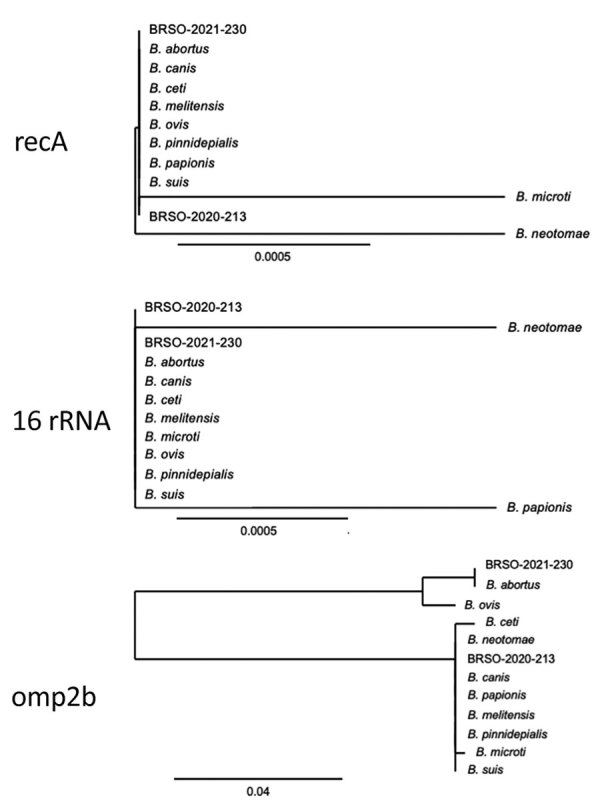
Phylogenetic placement of *Brucella* isolates BRSO-2020-213 and BRSO-2021-230 from 2 patients diagnosed with brucellosis in French Guiana, 2020, using alignments of *Brucella* genes. Sequences of the genes *recA*, 16S rRNA, and *omp2b* were aligned using the web tool Phylogeny (*17*) using the default parameters (“One Click” mode). Scale bar indicates number of substitutions per site.

Construction of a genome-based phylogenetic tree showed that BRSO-2020–213 and BRSO-2021-230 are very closely related and that both belong to the classical *Brucella* clade (Figure 3). Importantly, they did not cluster with any existing *Brucella* species. In silico multilocus sequencing typing, performed on GALAXY using CONTIGuator products as inputs (Florence Computation Biology Group, https://www.bio.unifi.it/p162.html), likewise showed that BRSO-2020-213 and BRSO-2021-230 do not belong to any known *Brucella* genotype. We performed alignments on 3 genes that are commonly used to discriminate *Brucella* species and reveal phylogenetic relationships among this genus: *recA*, 16S rRNA, and *omp2b* (*11*). We found the nucleotide sequences of *recA* to be 100% identical between BRSO-2020-213, BRSO-2021-230, and all other *Brucella* species (Figure 4), with the exception of *B. neotomae* and *B. microti*, for a which a single-nucleotide polymorphism was already described (*12*). We noted the same results for the 16S rRNA gene, with the exception of *B. neotomae* and *B. papionis*. The *omp2b* gene provided the most discriminant findings, with only 88% identity between BRSO-2020–213 and BRSO-2021-230. Altogether, these analyses indicate that BRSO-2020-213 and BRSO-2021-230 are 2 distinct isolates, representing a new species of *Brucella*.

## Discussion

Our report of 2 cases of human brucellosis in French Guiana follows a previous report of a single case of human brucellosis in this region, which described a 29-year-old man from Brazil who worked as a goldminer and became infected with *B. suis* biovar 1, likely because of contamination in a pork farm in the state of Maranhão, Brazil (A. Melzani et al., unpub. data). In the cases we report here, both patients were also citizens of Brazil who were smuggled into French Guiana, most likely for clandestine goldmining. Characterization of bacteria from our patients, however, revealed 2 distinct isolates representing a new species of *Brucella*, for which we propose the name *Brucella amazoniensis* sp. nov.

French Guiana spreads over 84,000 km^2^ and has a population of ≈300,000. The French territory is separated from Suriname by the Maroni River to the west and from Brazil by the Oyapock River to the east (Figure 1). Illegal gold mining is a growing practice in the territory, mainly in deep Amazon rainforest regions, and is carried out predominantly by clandestine immigrants from Brazil. Such clandestine goldminers represent a particularly vulnerable population who face precarious life conditions and high risks of exposure to wildlife and, thus, zoonotic pathogens (*13*–*15*).

We speculate a probable zoonotic transmission in the cases we report, most likely from a wild animal living in the Amazonian Forest. Both patients declared living “in the forest,” hunting and consuming “bush meat,” especially from swine. Although this new *Brucella* species seems genetically distinct from *B. suis*, it cannot be excluded that wild swine might be its animal reservoir. Two species of wild pigs live in this region and are commonly hunted: the collared peccary (*Pecari tajacu*) and white-lipped peccary (*Tayassu pecari*). We are conducting further research to determine if these suids, or other local wild Amazonian fauna, are the reservoir of this new *Brucella* species.

Medical practitioners should be aware of the existence of *Brucella* in this region of the world and that first-line laboratories perform simple *Brucella* serology tests (i.e., the Rose Bengal slide agglutination test or lateral flow assays) whenever symptoms or epidemiologic evaluations suggest possible brucellosis. When a gram-negative bacterium is isolated, positive reactions for urease, catalase, and oxidase is strongly indicative of *Brucella* spp., and suspect isolates should be manipulated using appropriate biosafety measures. Mass spectrometry can be used to confirm bacteriological identification (*16*) and should lead to further molecular characterization in specialized laboratories.

Appendix Additional information on a novel species of *Brucella* causing human brucellosis, French Guiana.
